# Dysbiosis in the Development of Type I Diabetes and Associated Complications: From Mechanisms to Targeted Gut Microbes Manipulation Therapies

**DOI:** 10.3390/ijms22052763

**Published:** 2021-03-09

**Authors:** Gratiela Gradisteanu Pircalabioru, Nicolae Corcionivoschi, Ozan Gundogdu, Mariana-Carmen Chifiriuc, Luminita Gabriela Marutescu, Bogdan Ispas, Octavian Savu

**Affiliations:** 1 Research Institute of University of Bucharest (ICUB), 300645 Bucharest, Romania; gratiela.gradisteanu@icub.unibuc.ro (G.G.P.); lumi.marutescu@gmail.com (L.G.M.); ispasbogdan26@gmail.com (B.I.); 2 Bacteriology Branch, Veterinary Sciences Division, Agri-Food and Biosciences Institute, Belfast BT9 5PX, UK; nicolae.corcionivoschi@afbini.gov.uk; 3 Faculty of Infectious & Tropical Diseases, London School of Hygiene and Tropical Medicine, Keppel Street, London WC1E 7HT, UK; Ozan.Gundogdu@lshtm.ac.uk; 4 Academy of Romanian Scientists, 050045 Bucharest, Romania; 5 “N.C. Paulescu” National Institute of Diabetes, Nutrition and Metabolic Diseases, 2nd District, 020042 Bucharest, Romania; savu.octavian@gmail.com; 6 Department of Doctoral School, “Carol Davila” University of Medicine and Pharmacy, 5th District, 050474 Bucharest, Romania

**Keywords:** microbiota, type 1 diabetes, microbiome, diet, dysbiosis

## Abstract

Globally, we are facing a worrying increase in type 1 diabetes mellitus (T1DM) incidence, with onset at younger age shedding light on the need to better understand the mechanisms of disease and step-up prevention. Given its implication in immune system development and regulation of metabolism, there is no surprise that the gut microbiota is a possible culprit behind T1DM pathogenesis. Additionally, microbiota manipulation by probiotics, prebiotics, dietary factors and microbiota transplantation can all modulate early host–microbiota interactions by enabling beneficial microbes with protective potential for individuals with T1DM or at high risk of developing T1DM. In this review, we discuss the challenges and perspectives of translating microbiome data into clinical practice. Nevertheless, this progress will only be possible if we focus our interest on developing numerous longitudinal, multicenter, interventional and double-blind randomized clinical trials to confirm their efficacy and safety of these therapeutic approaches.

## 1. Introduction

The human gastrointestinal tract (GIT) is home to almost 1000 different bacterial species, as well as viruses and fungi. Among these, the most abundant bacterial phyla are represented by *Bacteroidetes*, *Firmicutes*, *Proteobacteria and Actinobacteria* [1]. The microbiota population is stabilized by the age of three and is modulated by maternal diet composition, delivery mode, early nutrition (breast-milk versus formula feeding), intake of antibiotics and environmental factors (particularly hygiene level) [2].

The microbiota thrives in the nutrient-rich GIT and, in turn, provides several benefits for the host including digestion of dietary substrates, vitamin synthesis, modulation of the immune system, and protection against enteric pathogens via competition for nutrients and production of bacteriocins/other antimicrobial metabolites (i.e., hydrogen peroxide, lactic acid, etc.). A health-promoting microbiota is characterized by a certain level of microbial abundance and diversity that are not highly affected by transient perturbations caused by infections and antibiotic treatment and is also enriched in genes involved in fermentation and biosynthesis of short-chain fatty acids (SCFAs) [3].

Numerous studies from the last decade have shed light on the fact that microbiota is not just a collection of microorganisms that inhabit us, but a pivotal player for our health. Imbalances in the microbiota (also known as dysbiosis) are triggered by a multitude of factors including diet, pathological conditions (i.e., inflammatory bowel disease, cancer etc.), antibiotic intake, infections, stress and other environmental factors. In contrast, microbiota is an essential contributor to the development and progression of many diseases and therefore microbiota modulation holds promise as a potential therapeutic strategy. Globally, we are facing a worrying increase in type 1 diabetes mellitus (T1DM) incidence, with disease onset at younger age [4]. Research efforts have been made in the last few years and we have a general picture of the microbiome patterns linked to T1DM. Even though these studies reveal that taxonomic changes in the microbiota of T1DM patients are quite modest, a functional depletion in short chain fatty acids (SCFAs) production is commonly encountered across various studies and population groups [5].

Nevertheless, there is a paucity in studies correlating microbiota data with other host factors (i.e., metabolome, proteome, epigenetic markers, diet, etc.) as well as translating microbial diversity information into dietary/therapeutic intervention strategies. Within this review, we briefly highlight the microbiome features identified in T1DM patients together with the current intervention strategies targeting the gut microbiota and, last but not least, we discuss the challenges and perspectives of translating microbiome data into clinical practice.

## 2. Type 1 Diabetes Mellitus and the Microbiota—A Chicken and Egg Situation?

Diabetes mellitus (DM) is a heterogeneous disease, out of which type 1 DM (T1DM) is characterized by absolute lack of insulin that mainly results from autoimmune destruction of pancreatic beta cell mass (type 1A) [6]. In several cases only, in spite of a strong inheritance (type 1B), the cause of beta cell destruction is unknown [6]. As a population-wide screening for beta cell targeting autoimmunity is not yet established, the epidemiology of T1DM is based on either plasma glucose or HbA1c criteria, and classic symptoms of hyperglycemia [6]. The incidence rates of T1DM are increasing worldwide by 1.8–5% per year [7,8], and differ extensively upon geography and different ethnic populations [9], with highest occurrence in countries from Northern Europe and Canada [10]. The explanation for this variation remains unclear, although it may be related to specific risk factors, such as genetic susceptibility (i.e., population with certain types of HLA and multiple non-HLA loci) and environmental triggers (i.e., hygiene and infections) [11].

As T1DM does not occur in many subjects harboring genetic susceptibility clearly demonstrates the importance of other determinants responsible for actual T1DM increase in incidence [12]. The environmental exposure confers an additional risk for islet targeting autoimmunity [10,11]. Numerous environmental triggers (i.e., viral infections, gestational infections, childhood immunization, diet, especially the timing of first introduction of food) are thought to contribute to pancreatic beta cell destruction, but the mechanisms involved remain largely unknown.

However, recent data [13] support the essential contribution of exogenous triggers to rapid increase in T1DM incidence. In this scenario, the role of human microbiota in the development of (auto)immune diseases and T1DM has recently gain higher interest, as its close relations with the immune system [14] and chronic inflammation [15], and its profound and permanent changes with environmental factors [16]. Consecutively, the dysbiosis associated with (gut) microbiota modifications raises the need to better understand its significance as contemporary or causal phenomena in T1DM [17]. Evidence of a possible link between microbiota and T1DM has emerged from several animal studies. For instance, administration of single antibiotics (i.e., vancomycin) or cocktails of antibiotics triggered microbiota changes in non-obese diabetic (NOD) mice leading to accelerated or delayed disease progression [18,19,20,21,22].

The causative link microbiota-T1DM was proven in murine models. It was shown that T1DM development is in close relation with the host innate immunity. Hence, interaction of the commensal biota with the innate immune system impacted the onset and progression of T1DM. Toll-like receptors (TLRs), are crucial players in the innate immunity and intestinal homeostasis. Importantly, microorganisms can affect autoimmunity of T1D via signaling through TLR family receptors [23,24,25]. Interestingly, NOD mice deficient in MyD88, an adaptor molecule for TLR signaling, did not develop T1DM under conventional SPF (specific pathogen free) conditions but this protection was abolished under germ free conditions [26]. Moreover, T1DM incidence was reduced when MyD88 deficient mice were exposed to a predefined microbiota mixture, further highlighting the close connection between the microbiome and host immunity [26].

Another way by which bacteria can impact T1DM pathogenesis is through production of short chain fatty acids (SCFAs) such as butyrate, propionate and acetate. Several research reports linked abundance of butyrate-producing species with T1DM risk [27,28,29,30]. Studies show that butyrate may exert epigenetic effects with a paramount role in T1DM such as histone acetylation in the promoter of the *Foxp3* locus responsible for the differentiation of regulatory T cells or inhibition of histone deacetylases in macrophages [31,32,33]. However, current data does not elucidate whether SCFAs induce tolerance by modulating either host microbiome or inflammatory response [34].

Opposite to most previous reports [35,36], studies from recent years have shown that in the case of T1DM, microbiota changes occur prior to systemic signs of islet autoimmunity [37,38]. The reason for this shift may resides in that the microbiota modifications were mostly detected only by gene analysis of 16S rRNA, which may not identify specific structural and/or functional characteristics potentially involved in disease progression. Later studies addressed this issue both by a specific design allowing to control for all known factors interfering for T1DM susceptibility and microbiome characteristics [38], and by longitudinal metagenomic sequencing of stool samples [37]. Regardless of geographical location, the T1DM environmental niche was shown to harbor a proinflammatory environment colonized by higher level of Bacteroidetes and low abundance of Firmicutes [39]. It has been suggested that the reduction in Firmicutes may be detrimental for the host because this phylum reunites many producers of the SCFA butyrate, known to be involved in intestinal homeostasis. Moreover, the Bacteroidetes phylum is comprised, among others, of *Bacteroides* and *Prevotella* strains [39]. Many studies have shown that T1DM is dominated by *Bacteroides*, a taxa correlated with intestinal inflammation whereas *Prevotella*, believed to be protective, is reduced [40].

Regardless of the great variability found in T1DM associated microbiota, several studies consistently reported that *Bacteroides* was linked to T1DM development [27,28,41,42,43,44]. Indeed, species belonging to this genus can ferment glucose and lactate to propionate, acetate, and succinate [45] while not being able to generate butyrate [46]. Butyrate is a crucial metabolite for intestinal homeostasis that stimulates mucin synthesis [47] and can also decrease gut permeability by enabling the assembly of tight junctions (TJ) [48]. In addition lactate producing bacteria, including some probiotic strains (*Lactobacillus rhamnosus* and *L. reuteri* [49], *L. johnsonii* N6.2 [50], *L. plantarum* [51], *Bifidobacterium lactis* [52] may synthetise butyrate [53] and, thus, strengthen the intestinal barrier function.

All these findings paint the picture for microbiota involvement in T1DM pathogenesis but, nevertheless, conclusions from animal studies need to be taken with caution [54]. Some studies reported that alterations in microbiome composition were evident prior to T1DM development [27,28,36,55]. However, these were prospective studies that presented a timeframe of disease progression linked to microbial dysbiosis, and to address the issue of a causal contribution of the microbiota in this ailment, intervention studies are needed.

Knowledge regarding the involvement of probiotics in this equation is still scarce. Nevertheless, early administration of probiotics during the first four weeks of life diminished the risk of beta cell autoimmunity in subjects genetically susceptible to T1DM when compared to those that received no probiotics [56]. Administration of the prebiotic oligofructose-enriched inulin in a single-center, randomized, double-blind, placebo-controlled pilot study in children with T1DM was shown to improve beta cell function and intestinal permeability. Nevertheless, prebiotic administration did not improve glycaemic control likely due to the small sample size analyzed [57]. Even though interventional human studies suggest a microbial involvement in T1DM, a causal relationship still cannot be inferred due to the lack of large-scale prospective studies that clearly demonstrate that microbiome changes alter T1DM risk. Future randomized controlled studies in large cohorts are still necessary. For instance, there are no human studies to address the link between antibiotic intake—microbiota changes—T1DM or between FMT-microbiota-T1DM.

## 3. Translational Applications—Moving from Fundamental Research to Improved Diagnosis and Therapeutic Strategies in T1DM

**Figure 1:**
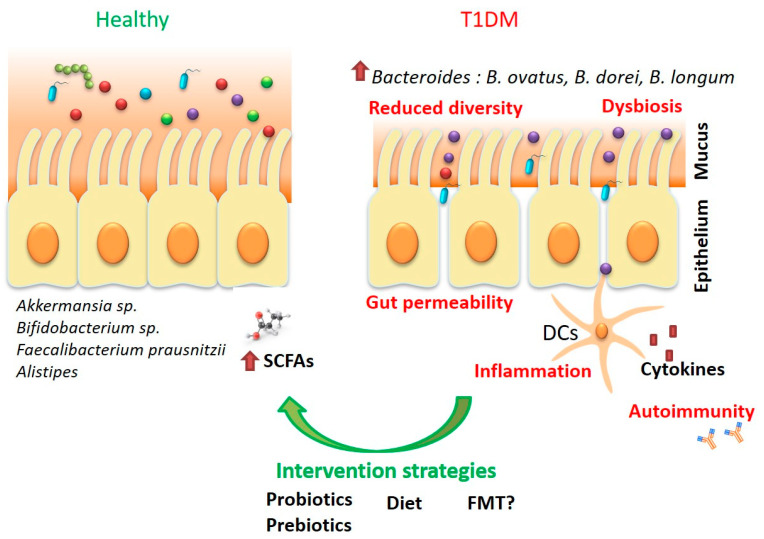
Gut microbiota and T1DM. T1DM patients harbor a microbiota with reduced diversity enriched in *Bacteroides* species (*B. dorei*, *B. ovatus*, *B. logum*). The gut environment in T1DM is characterized by increased gut permeability, disrupted mucus barrier, inflammation and low production of SCFAs (particularly butyrate).

As our knowledge in the microbiome field expands, it is becoming more evident that reshaping the gut microbiota through various strategies (i.e., diet, probiotics, prebiotics, fecal transplant) holds promise in T1DM therapy in terms of halting disease progression and even prevention (Figure 1). Personalized changes in the host microbiota, customized in accordance to the host genetic background may be a powerful new approach in prevention and treatment.

### 3.1. Diet

It is well established that microbiota composition is highly dependent on host diet [58]. In subjects with autoimmune diseases, microbiota suffers significant differences compared with healthy persons [30,59,60]. The link between diet inductors of gut dysbiosis (i.e., fat from bovine milk, protein from fresh milk or gluten) and subsequent development of T1DM has been suggested in several human studies [61,62,63]. The impact of dietary style on intestinal microbiota and the occurrence of T1DM is furthermore demonstrated by studies involving second generation of immigrants coming to Sweden [64]. Post-immigration occurrence of metabolic diseases in relation with diet modifications were also reported in migrants coming to U.S [65].

Experiments on mice have shown that a high-protein diet leads to an increased level of IGF-1 (insulin-like growth factor 1) which is correlated with a high risk of developing diabetes. Studies on mice fed with solid animal fats have revealed a greater abundance of species of the genus *Bacteroides* and *Bilophila*, while in mice fed with fish oil dominated the *Bifidobacterium*, *Adlercreutzia*, *Lactobacillus*, *Streptococcus* genera and *A. muciniphila*. The mice fed with lard developed inflammation in the white adipose tissue, low insulin sensitivity, TLR (Toll-like receptor) activation, as opposed to those fed with fish oil [66]. The artificial sweeteners such as saccharin, sucralose, and aspartame have been proposed as an alternative to natural sugar and promoted as healthy and without calories, although they are quite controversial and the opinions and expert opinion is divided. The consumption of artificial sweeteners has many consequences such as structural and functional alterations of the intestinal microbiome, causes glucose intolerance and determines a predisposition to diabetes [67].

Data from human and animal studies suggest that early exposure to foreign food antigens such as bovine insulin and gluten may be involved in β-cell immunity [68,69]. Therefore, it is not surprising that diet is also an important contributor to T1DM onset and progression. As previously mentioned, a leaky gut barrier is commonly encountered in T1DM [41]. Gluten was shown to harbor various effects in the body, starting from the intestine where it may alter microbiota composition triggering enteropathy and intestinal permeability [70]. Moreover, these effects can be modulated by a gluten free diet [71]. As revealed by animal studies, a gluten free diet may dampen the innate and adaptive immune system proving to be beneficial for the host [71]. In human T1DM, several intervention strategies have been proposed including gluten exclusion during pregnancy combined with early gluten introduction, mucosal tolerance induction to gluten as well as gluten free diets [71]. Gluten-based diets were linked to gut permeability alterations via microbiome modulation [72]. For example, taxa associated to a gluten free diet (i.e., *Akkermansia* sp.) were reported to impact gut permeability by regulating tight junction proteins and to be protective in case of T1DM [73].

Both animal and human studies reveal the fact that early exposure to gluten (<3 months in humans) raises the risk to develop islet autoimmunity [74,75]. In patients with genetic predisposition, who are positive for HLA DQ2/DQ8, gluten activates the specific T lymphocytes, leading to mucosal destruction, hyperplasia of intestinal crypts, as well as subtotal or total vilositary atrophy [76].

Though, in both celiac disease and T1DM, the effects of gluten free diet are still controversial. Hence, it was reported that in children with established T1DM and concomitant biopsy diagnosis of coeliac disease over a 10-year period, gluten-free diet significantly improved both weight, height, and BMI adjusted for age, and improved diabetes control by reducing daily insulin doses [77]. In NOD mice, gluten free diet prevents diabetes by reducing the number of caecal bacteria [78]. However, other reports did not find a significant effect [79,80].

Cow’s milk is another dietary factor believed to be involved in T1DM but this aspect is a subject of controversy. While some studies highlight that bovine milk proteins may be a culprit in mounting a humoral immune response leading to disease onset [81], other studies found no differences [82] or even implied that cow’s milk formula has a beneficial effect against T1DM [83]. Moreover, cow’s milk formula containing bovine insulin was shown to trigger an autoimmune response to insulin. In addition, a subsequent study (Finnish Dietary Intervention Trial for the Prevention of T1DM) reported that milk formula without bovine insulin reduced the risk of β-cells autoimmunity [84].

Breast milk induces dose dependent changes in the infant microbiota that correlate with specific bacteria (i.e., *Bifidobacterium* and *Lactobacillus*) transferred from maternal microbiota [85,86]. As for gluten, murine studies suggested that for breast milk the timing of introduction is essential for an efficient microbiome regulation of the infant’s immune system [87]. Hence, it has been shown that infants that were breastfed only during first 6 months of life have developed a distinct pattern of microbiota [1]. Moreover, it is suggested that breast milk protects against T1DM occurrence in children with genetic predisposition [88].

Recent data [89] have shown that the occurrence of islet autoimmunity and the progression towards T1DM in genetically predisposed children was not influenced either by the age of solid food introduction or by the duration of breastfeeding. These results support previous observation showing that breastfeeding alone may interfere with T1DM occurrence [90,91]. Nevertheless, how and to what extent dietary factors influence T1DM pathophysiology is an important aspect that awaits future research.

Permanent dietary changes can trigger the enrichment of beneficial taxa shaping the intestinal homeostasis, but we have to highlight the paramount importance of the patient ability to adhere to certain diets. Moreover, one major challenge in this field is to decipher the high variability in individual responses to food intake. People differ greatly in their responses to diet intervention due to a wide array of factors some of which are non-food-specific factors (sleep, meal timing and activity). Hence, dietary interventions hold promise in improving host health but their implementation should be carefully optimized using rigorous prediction algorithms.

### 3.2. Probiotics—The Promises and the Unmet Needs

Probiotics are microorganisms (bacteria, fungi) commonly regarded as safe that, when administered to a subject, promote a wide range of beneficial effects including modulation of gut microbiota and immunomodulation.

Most of the progress in understanding and developing microbiome therapeutics has emerged from rodent models. However, rodents and humans harbor fundamentally different microbiomes, so making the transition from one to another needs to be performed with caution. Several probiotics are currently being used in clinical trials to correct the dysbiosis linked toT1DM [92].

Besides the well-known lactobacilli, promising probiotics that are potent SCFA producers (especially butyrate) such as *Roseburia intestinalis*, *Eubacterium halli* and *Faecalibacterium* spp. can be employed in clinical trials [93]. For instance, probiotics such as *B. lactis* Bb12L and *L. rhamnosus* GG are currently being investigated for their protective role in a clinical study in children with newly diagnosed T1DM [72]. Notably, probiotic administration (lactobacilli and bifidobacterial species) early in life was significantly correlated with a reduced autoimmunity risk in subjects harboring an HLA-DR3/4 genotype [56]. Currently, several ongoing clinical studies are investigating the role of probiotics such as *L. johnsonii*, *L. plantarum*, *L. salivarius AP-32*, *VSL#3*, and *B. animalis subsp. lactis CP-9* in T1DM management (NCT03880760, NCT03423589, NCT03423589, NCT04141761, NCT03961854, NCT04335656). Most of these clinical studies are in the recruitment phase but, once completed, they may provide a better picture regarding the impact of probiotic supplementation on metabolic profile (blood glucose, HbA1c), systemic inflammation, host transcriptome, gut barrier function, and microbiome profile.

Even though a series of recent papers have addressed the various microbiota patterns linked to diabetes, still there is research needed to assess the impact of microbiota manipulation on development of diabetes complications [5,17,40,54,72,94,95,96,97]. Specifically, the complications triggered by diabetes can be either microvascular (nephropathy, retinopathy, and neuropathy) or macrovascular (i.e., cardiovascular disease, peripheral vascular disease, cerebrovascular accidents). In the last few years, several research groups have investigated this aspect particularly in the case of diabetic nephropathy. Indeed, gut microbiota is impaired in chronic kidney disease likely due to elevated uremia which halts intestinal barrier function triggering intestinal inflammation which in turn may disturb the renal parameters. Therefore, manipulation of gut microbiota may positively modulate the renal profiles in these patients. The results emerging from studies of probiotic use in diabetes complications are summarized in Table 1. Most of the microorganisms used in these studies belonged to the *Lactobacillus* and *Bifidobacterium* genra whereas the dosage ranged from 2 × 10^7^ to 6 × 10^10^ CFU/g. The form of the probiotics varied across the studies (capsules, soy milk, sachets, kefir and honey). Most studies demonstrated the benefits of probiotic supplementation on diminishing inflammation, oxidative stress and on the amelioration of renal function biomarkers in patients with diabetic nephropathy.

Even though there is a high amount of research being done in the field of probiotics and their positive effects on various ailments, there are still some challenges left. Importantly, a probiotic strain needs to establish itself within the intestinal niche in order to elicit its host beneficial effects. However, to do this, the probiotic strain may have to outcompete the indigenous microbiota while being resistant to the host antimicrobial peptides. Since we know that the host microbiota varies from individual to individual, we can say that probiotic outcomes are individualized and, to some extent, unpredictable. Nevertheless, some studies advocate that establishment of a probiotic within the gut microbiota is not mandatory to elicit a beneficial effect. Therefore, while probiotics may be transient, they may modulate the microbiome via the production of beneficial metabolites including SCFAs.

Moreover, studies and clinical trials employing probiotics for treatment of T1DM (or any other disease for that matter) need to provide information regarding patient details such as age, gender, geographical location, comorbidities, medication taken, and nutrition as well as the impact of probiotics on the host metabolome (particularly SCFAs, bile acids) and immune system. Only such randomized, placebo-controlled studies may help us decipher the gut-microbiome interplay and may guide us to identify which patients may benefit from a certain probiotic or not.

### 3.3. Prebiotics—Potential Adjuvants in Glycemic Control

Prebiotics, defined as non-digestible food ingredients that modulate the microbiota by serving as nutritional substrates for the growth and multiplication of probiotics, also hold therapeutic potential. Inulin, human milk oligosaccharides, and lactulose are prebiotics that boost the growth of various probiotic strains including bifidobacteria. Current management of T1DM is based on multiple daily subcutaneous insulin injections or infusion and frequent glycaemic monitorisation. Unfortunately, it is still challenging to reach optimal glycemic control in these patients. Hence, in this scenario, adjunctive oral supplements in the shape of prebiotics may help improve glycemic control. Within this line of thought, a recent study by Ho et al. used oligofructose-enriched inulin in a randomized, placebo-controlled trial performed in T1DM children. Treatment with oligofructose enriched inulin was shown to harbor beneficial effects in T1DM children by significantly increasing C-peptide levels [57]. Moreover, a randomized, placebo-controlled trial recently showed that prebiotics may positively impact glycaemic control through direct interventions on gut microbiota by prebiotic administration and subsequent reducing of intestinal permeability, thereby improving insulin sensitivity in young T1DM patients [107]. This study confirmed in children with established T1DM previous observation on obese mice [108] that reduced gut permeability involves an increased level of glucagon like peptide (GLP)-2.

Most of the studies addressing the impact of prebiotics on T1DM have been performed on animal models and so far, not many clinical trials in humans exist. Notably, an acetylated and butyrylated form of high amylose maize starch (HAMS-AB) that was shown to increase SCFAs production and to be safe and effective in disease prevention in T1DM mouse models is currently being used in a phase 3 clinical trial (NCT04114357) in children and adolescents with recently diagnosed T1DM.

### 3.4. Faecal Microbiota Transplant—Solution or Potential Problem?

Host microbiota can be also modified by faecal microbiota transplant (FMT), which involves the transfer of a ‘healthy’ microbiome into a recipient with dysbiosis with the purpose to restore gut homeostasis. FMT is being used successfully in treating *Clostridium difficile* infection, however its success in treating T1DM is largely unknown.

A recent study by de Groot et al. showed that autologous (i.e., from T1DM patients) but not homologus (i.e., from healthy donors) FMT of colon-derived microbiota into the intestine of patients with new onset T1DM lead to a prolonged residual beta cell function. One possible explanation proposed by authors is that the autologous FMT is more immunologically compatible with the host. The assumption is in line with the results of the study showing that the preservation of beta cell function by autologous FMT is T cell mediated, as CD4+ CXCR3+ and CD8+ CXCR3+ T cells were decreased differentially in the responders.

In addition, the study identifies several taxa with therapeutic potential including duodenal *Prevotella* spp. and *S. oralis* as well as faecal *Desulfovibrio piger* and *Bacteroides stercoris* [109]. Both *Prevotella* spp. and *S. oralis* were negatively correlated with the most important fasting plasma metabolite that changed upon FMT and with fasting C-peptide. *Bacteroides stercoris* correlated positively with *Desulfovibrio piger* while the latter correlated positively with another fasting plasma metabolite that changed upon FMT and with fasting C-peptide. Moreover, *Desulfovibrio piger* was negatively correlated with CD4+ CXCR3+ and CD8+ CXCR3+ T cells. These results suggest that taxa that changed upon FMT in subjects with T1DM has beneficial effects either by preserving beta cell function or by suppressing autoimmunity.

Nevertheless, one has to bear in mind the fact that while probiotic administration transfers few microbial species to the recipient gut, FMT conveys a mix of various microorganisms into the host, out of which some may possibly be hazardous for the recipient host. Thus, in order to consider FMT as a potent therapeutic approach, composition of the faecal microbiota for transplantation needs to be standardized prior to administration. Importantly, we still do not fully know the profile of the “perfect” donor and other problematic factors including sustainability of the procedure and risk of infection need to be dealt with.

## 4. Omic Technologies—From Bench to Bedside

Multi omics approaches are an area of active investigation that may ultimately offer opportunity for personalized treatment for many ailments. Importantly, most of the omics-based studies on diabetes reported so far used mainly patient serum samples. Nevertheless, it is important to also look into other types of samples (i.e., fecal samples) and to correlate multi-omic approaches with the gut microbiota. Only in this way we can reconstitute the flow of information from disease parameters to causes of disease (genetic or environmental), functional consequences and treatment opportunities.

Several research studies exist regarding multi omic analysis in case of diabetes but most of them are focused on type 2 diabetes rather than T1DM [110]. Nevertheless, transcriptomics analysis done by using microRNA (miRNA) microarray, followed by qRT-PCR (quantitative Real Time PCR) validation showed some specific features linked to T1DM. Thus, several miRNAs in peripheral blood mononuclear cells were reported to be specific to T1DM: miR-103, let-7, miR-1260, miR-130a, miR-1274, miR-150 miR-720, miR-193a-5p, miR-20b, miR-16-5p, miR-133a-3p, miR-142-5p, miR-409-5p, miR-501-3p, miR-486-5p, miR-145-3p, miR-3150-3p and miR-1271-5p, with some of them being linked to retinopathy and nephropathy [111,112,113].

Proteomic approaches have been used to elucidate the mechanisms of beta-cell dysfunction in diabetes. Thus, using proteomics diagnostic tools, six new T1DM autoantibodies have been identified. More specifically, these autoantibodies are against receptor type N2 (PTPRN2), protein tyrosine phosphatase, nucleoporin 50, mutL homolog 1 (MLH1), peptidyl-prolyl cis-trans isomerase-like 2 (PPIL2), mitochondrial translational initiation factor 3 (MTIF3) and pyroglutamylated RFamide peptide receptor (QRFPR) [114]. Besides autoantibodies, serum adiposity proteins and immune molecules have been linked to T1DM but their validity has yet to be studied through proteomics [115].

Even though metabolomics data may have a powerful impact on T1DM management, current therapeutic approaches revolve around targeting high glucose levels and glycosylated hemoglobin. It was reported that patients with diabetes (both type 1 and type 2) harbored elevated levels of aromatic and branched-chain essential amino acids [116] which are produced by members of the microbiota such as *Escherichia coli* [117]. Microbiota is closely related to the metabolomic profile as it was recently shown for type 2 diabetes in an elegant study by Nuli et al. (2019) [118]. Faecal metabolites such as protorifamycin I, matricin, and epothilone A were negatively correlated with *Actinobacteria*. A similar testing for the T1DM fecal metabolome awaits investigation [118].

Several reports have also linked T1DM autoimmune development with lipidomic changes. Hence, changes in the metabolomic profiles of various classes of lipids, such as triglycerides, plasma phospholipids, sphingolipids, glycerophospholipids, and cholesterol esters, within the context of T1D have been reported [116,119,120,121].

Since the management of all types of diabetes comprises of dietary and lifestyle interventions, future work aiming to analyze how diet impacts metabolites and the microbiome is essential.

The main advantage of using omics-based technologies is the fact they provide simultaneous detection of multiple molecules, helping to better understand and potentially treat disease. Nevertheless, there is still a lot we need to understand in order to apply a personalized treatment approach in T1DM. It is imperative to further standardize and automate these methods of analysis particularly in the case of metabolomics and proteomics in order to make efficient and reproducible high-throughput analyses. Despite the great promise of integrating big data multiomics into patient treatment, there are limitations as well as ethical considerations currently halting its large scale implementation. Beside the logistic concerns surrounding implementation of these technologies, these omic approaches are very costly and, moreover, there is a general reluctance to change in health-care systems. Unfortunately, though, there is also a fundamental lack of recognition by funding agencies of the potential for omic-guided care to improve disease outcome.

**Figure 2:**
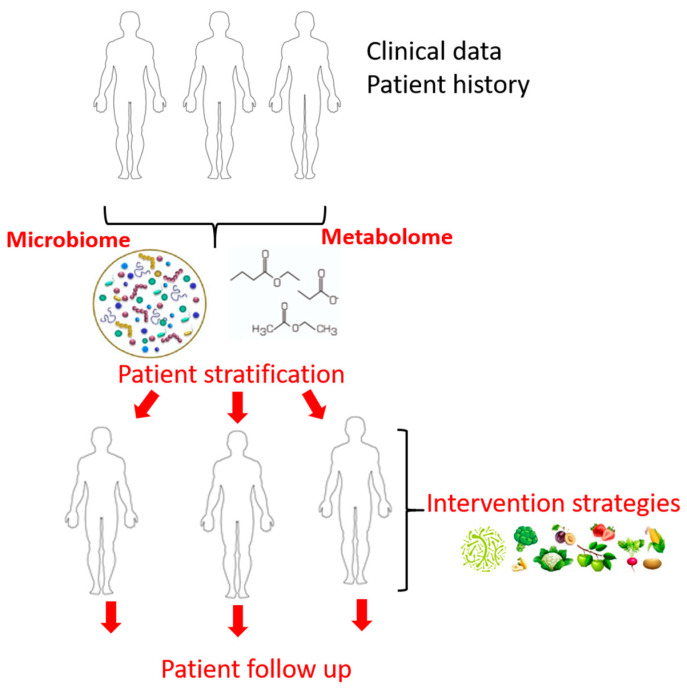
Proposed personalized therapeutic approaches in T1DM.

We show here that several methods to tailor the host microbiota have good potential in improving T1DM outcome. We believe that combining traditional sources of medical information (i.e., laboratory workup, patient history) with data emerging from omic technologies (i.e., microbiome, metabolome) will enable patient stratification subsequently aiding the clinician to choose the appropriate intervention strategies (diet, probiotics, fecal transplant etc.). It is imperative to make the transition from the lab to the clinic so that we can help deliver precision medicine to individual patients (Figure 2). Moreover, a patient follow-up after the intervention is crucial.

## 5. Conclusions

Clinical management of diabetes involves strategies targeting modifiable risk factors that aim to reduce the development of specific complications. As lack of endogenous insulin is the key element in pathogenesis of T1DM, exogenous insulin therapy is of outmost importance. However, high variability occurs in response to classical therapeutic interventions. Hence, clinical approaches in diabetes as well as in other ailments need to switch from “one size fits all” to personalized medicine. A better understanding of the unique characteristics of each patient will explain the underlying causes for differential pharmacological responses and will improve treatment options. Knowledge gained from microbiome research is a promising step in T1DM field. Even though the studies presented within this review offer a picture regarding the microbiome thriving in the T1DM gut, they generally targeted white populations with early-onset disease so there are still many questions awaiting.

Nevertheless, by targeting the microbiota, we will be able to more precisely characterize and treat diabetes. Probiotics, prebiotics, dietary factors and microbiota transplantation can all modulate early host–microbiota interactions by enabling beneficial microbes with protective potential for individuals with T1DM or at high risk of developing T1DM. Nevertheless, this progress will only be possible if we focus our interest on developing numerous longitudinal, multicenter, interventional and double-blind randomized clinical trials to confirm their efficacy and safety of these therapeutic approaches.

Moreover, the advent of omics profiling technologies has a huge impact in identifying unique biological signatures to allow personalized treatments in diabetes. Importantly, omic technologies need be scaled up and made available to many individuals because currently, many of these tools used in precision medicine are expensive and not accessible in many parts of the world. Future research studies need to better characterize health-associated taxa in correlation with their functional features (i.e., SCFAs production) and their delivery to a recipient host. Importantly, the patient’s individual microbial profiles should be characterized before employing personalized therapeutic approaches.

## Figures and Tables

**Table 1:** Studies of probiotic use in diabetes complications.

Study Group Details	Treatment	Outcome	Reference
Diabetic nephropathy, 44 subjects probiotic (*n* = 22) placebo (*n* = 22)	Soy milk containing *L. plantarum* A7 adminsitered for 8 weeks	Significant impact on lipid profile and glomerular function	Abbasi et al., 2017 [98]
Diabetic nephropathy, 136 subjects probiotic (*n* = 68) placebo (*n* = 68)	*L.acidophilus*, *L. casei*, *L. lactis*, *B. bifidum*, *B. infantis*, *B. longum* and with a daily dose of 6 × 10^10^ 12 weeks	The urea levels significantly declined in the probiotic group while her parameters of renal profile as well as liver function tests remained unchanged	Firouzi et al., 2015 [99]
Diabetic nephropathy 60 subjects probiotic (*n* = 30) placebo (*n* = 30)	8 × 10^9^ CFU day^−1^ of probiotic supplements containing *L.* *acidophilus* strain ZT-L1, *B. bifidum* strain ZT-B1, *L.* *reuteri* strain ZT-Lre, and *L. fermentum* strain ZT-L3 (each 2 × 10^9^) 12 weeks	Probiotics supplementation for 12 weeks had beneficial effects on glycemic control and markers of cardio-metabolic risk. It may confer advantageous therapeutic potential for patients with diabetic nephropathy	Mafi et al., 2018 [100]
Diabetic nephropathy, probiotic (*n* = 30) placebo (*n* = 30)	*L. acidophilus*, *L. casei* and *B. bifidum* 12 weeks	Probiotic supplementation for 12 weeks among diabetic HD patients had beneficial effects on the parameters of glucose homeostasis and a few biomarkers of inflammation and oxidative stress	Soleimani et al., 2016 [101]
Diabetic nephropathy 60 subjects probiotic (*n* = 30) placebo (*n* = 30)	*Bacillus coagulans* T11 (IBRCM10791) (10^8^ CFU/g) 12 weeks	Probiotic honey consumption lead to improved insulin metabolism, total-/HDLcholesterol, plasma MDA levels, and serum hs-CRP, and but did not affect other metabolic profiles	Mazruei et al., 2019 [102]
Diabetic nephropathy, *n* = 48 48 subjects probiotic (*n* = 24) placebo (*n* = 24)	200 mL/day probiotic (*L. plantarum* A7 strain) soy milk in the intervention group or soy milk in the control group 8 weeks	DN participants in the probiotic soy milk group had higher levels of GSH compared to those in the soy milk group. Significantly increased levels of glutathione reductase and glutathione peroxidase were reported for the probiotic group	Miraghajani et al., 2017 [103]
Diabetic retinopathy Animal study	*L. rhamnosus* administration 4 months	Probiotic administration reduced the intraocular pressure in diabetic mice	Home, 2020 [104]
Diabetes complicated by coronary heart disease robiotic supplements (*n* = 30) or placebo (*n* = 30) for 12 weeks.	Oral administration of 2.5 × 10^9^ CFU/g probiotic containing *B. bifidum*, *B. lactis*, *L. acidophilus*, *L. brevis*, *L. casei*, *L. salivarius*, *L. lactis* and *L. lactis* twice a day 12 weeks	Probiotic supplementation significantly decreased fasting plasma glucose, insulin resistance and total-/HDL-cholesterol ratio. Probiotic administration significantly increased insulin sensitivity and HDL-cholesterol levels compared to the placebo group	Raygan et al., 2018 [105]
Diabetic mice	*L. paracasei* secreting Angiotensin-(1-7) 1 × 10^9^ CFU 8 weeks	Probiotic treatment treatment significantly lowered apoptotic cell death in kidney, improved diabetes-induced collagen deposits in the glomerular tuft and the tubular epithelia in diabetic mice. LP-A administration also significantly improved retinal gliosis, neuronal cell death inflammation, and loss of retinal vascular capillaries.	Li et al., 2018 [106]

## Data Availability

Not applicable.
